# Is serum thymidine kinase 1 a prognostic biomarker in primary tumor location of colorectal carcinomas?

**DOI:** 10.1007/s12672-023-00614-5

**Published:** 2023-02-17

**Authors:** Yujing Fang, Sven Skog, Qingjian Ou, Zhiheng Chen, Senbo Liu, Ailian Hei, Jin Li, Ji Zhou, Ellen He, Desen Wan

**Affiliations:** 1 Department of Colorectal Surgery, State Key Laboratory of Oncology in South China, Collaborative Innovation Centre for Cancer Medicine, Sun Yassin University Cancer Centre, Guangzhou, Guangdong 510060 People’s Republic of China; 2 Department of Experimental Research, State Key Laboratory of Oncology in South China, Collaborative Innovation Centre for Cancer Medicine, Sun Yassin University Cancer Centre, Guangzhou, 510060 Guangdong People’s Republic of China; 3 Department of Medicine, Shenzhen Ellen-Sven Precision Medicine Institute, 3rd Floor, Building 1, Guanlan Street, Longhua District, Shenzhen, 518110 Guangdong People’s Republic of China; 4 Management Centre, Third Xiangyan Hospital, Central South University, Changsha, 410013 People’s Republic of China

**Keywords:** Serum thymidine kinase 1 protein concentration (STK1p), CEA, CA19.9, Colorectal carcinoma (CRC), Left colon carcinoma (L-CC), Right colon carcinoma (R-CC), Rectal carcinoma (RC), Overall survival (OS)

## Abstract

**Aim:**

To assess whether serum thymidine kinase 1 (STK1p), CEA and CA19.9 can be used as prognostic biomarkers in the primary tumor location (PTL) of colorectal carcinoma (CRC). Additional clinical factors of TNM stage, pathological grade, age and sex were also included.

**Methods:**

STK1p was determined by an ECL-dot-blot assay, and CEA/CA19.9 was determined by an automatic electrochemiluminescence analyzer in a retrospective presurgery of right-colon carcinoma (R-CC, n = 90), left-colon carcinoma (L-CC, n = 128) and rectal carcinoma (RC, n = 270). Prognostic factors were evaluated by COX and overall survival (OS).

**Results:**

The multivariate-COX and OS in relation to the prognostic factors of PTL in CRC were different and complex. An elevated STK1p value was significantly associated with poor OS in RC (P = 0.002) and L-CC (P = 0.037) but not in R-CC (P > 0.05). Elevated CEA (P≈.000) and CA19.9 (P≈.000) were significantly associated with poor OS in RC but not in L-CC and R-CC. Multivariate-COX showed that STK1p (P = 0.02, HR = 1.779, 95%CI 1.30–7.582), CEA (P = 0.001, HR = 2.052, 95%CI 1.320–3.189), CA19.9 (P≈.000, HR = 2.574, 95%CI 1.592–4.162) and TNM-stage (P≈.000, HR = 2.368, 95%CI 1.518–3.694) were independent prognostic factors in RC, while TNM-stage was an independent prognostic factor only in R-CC (P = 0.011, HR = 3.139, 95% CI 1.30–7.582) and L-CC (P≈.000, HR = 4.168, 95%CI 1.980–8.852). Moreover, elevated STK1p was significantly more sensitive (P < .001) for predicting mortality than CEA and CA19.9. No correlation was found between STK1p, CEA or AFP.

**Conclusion:**

Combining TNM stage and suitable biomarkers, STK1p provides further reliable information on the survival of PTL of CRC.

## Introduction

Epidemiological data released by the Global Cancer Observatory (GCO) for 2020 showed that colorectal cancer (CRC) is the third most common cancer in the world according to incidence. Nearly 1.93 million new cases and approximately 0.94 million mortalities are expected in 2020 [1]. Furthermore, in the next 15 years, a 60% increase is predicted. This means that approximately 13 million patients will be estimated to die from CRC by 2030 [2]. In China, the increase in CRC deaths would primarily result from the rapid aging of the population [3]. Regarding the period for colorectal carcinoma, it was suggested that ten or fifteen years would be needed from mutation accumulation to invasive malignancy. These facts provide a possible time window to screen, detect and remove premalignant lesions early, which would contribute to improving overall survival [2, 4]. Currently, the 5-year survival rate has reached more than 60% among CRC patients at the early stage. However, in clinical practice, more than 50% of CRC patients are diagnosed with CRC at a late stage. Meanwhile, the 5-year survival rate declines to 10% in those cases [5].

Colorectal cancer (CRC) describes both colon cancer (CC) and rectal cancer (RC) because both CC and RC affect the large intestine. The colon and rectum have the same anatomical structure, which consists of the mucosa, muscular layer, and serosa, and the same functions, such as stool concentration, fluid resorption, stool transportation and excretion, and a similar histology [6]. However, recently, the primary tumor location (PTL) of CRC has drawn increasing attention to prove the significant difference between right-colon carcinoma (R-CC) and left-colon carcinoma (L-CC) in terms of epidemiological, clinical, and histological characteristics. It was found that R-CC seems to present a more advanced stage and more aggressive recurrence than L-CC [6–8] and RC [7–9]. The contributors include more mutations of the C-K-RAS proto-oncogene, mucinous type of tumor, older age, female sex and comorbidities [10], which may result in R-CC being more likely to relapse after surgery. R-CC patients may have a better prognosis after curative resection in TNM stage I-II and a worse survival after recurrence in TNM stage III [11, 12].

Developing appropriate serum prognostic indicators for preoperative stage and combining those indicators with the staging system would improve the accuracy of survival predictions. Currently, carcinoembryonic antigen (CEA) is commonly used in patients with CRC, alone or in combination with other biomarkers, such as cancer antigen 19.9 (CA19.9), cancer antigen 125 (CA125), cancer antigen 72–4 (CA72-4) and serum ferritin (SF) [13]. CEA was reported to be an independent prognostic factor for CRC [14].

It is necessary to search for serum proliferating markers, using a noninvasive method, [15] for the assessment of early dysplasia and the prognosis of R-CC, L-CC and RC. Human thymidine kinase 1 (HTK1) is an enzyme that catalyzes the conversion of thymidine (dThd) to thymidine monophosphate (dTMP) in the pyrimidine salvage pathway. HTK1 is mainly involved in DNA synthesis during the S phase of the cell cycle and was used to evaluate the proliferation rate in the 1950s [16–18]. It has been reported that a chicken anti-HTK1-IgY-poly-antibody (TK1-IgY-pAb) raised against the C-terminal peptide 195–225 of HTK1 can provide a sensitive, specific assay for serum thymidine kinase 1 protein (STK1p) measurement [17, 19]. Moreover, STK1p was proven to be an independent prognostic factor for recurrent breast cancer patients [20, 21], non-Hodgkin’s lymphoma patients [22], chronic lymphocytic leukemia [23], overall survival of non-small cell lung carcinoma [24], and a potential biomarker for the early detection of tumor risk progression in a health screening setting [17, 19, 25–27]. Recently, a meta-analysis of CRCs showed that STK1p values were significantly different between tumor-free individuals (n = 1887), colorectal adenoma poly-polyp/dysplasia patients (n = 1165), and CRC patients (n = 2251). The level of STK1p significantly increased in the order of tumor free < adenoma polyp/dysplasia < CRC TNM stage I-III (p < 0.0001) [28].

The purpose of this study was to investigate, for the first time, whether STK1p is a more useful prognostic marker in primary tumor location (PTL) of CRC than CEA and CA19.9. In addition, we focused on possible prognostic differences for R-CC and L-CC and for RC in presurgery primary patients by assessing multivariate-COX and overall survival (OS). Our study was performed according to the rules of the Reporting Recommendations for Tumor Marker Prognostic Studies (REMARK) [29].

## Results

### Characteristics of TK1-IgY-pAb

**Fig. 1:**
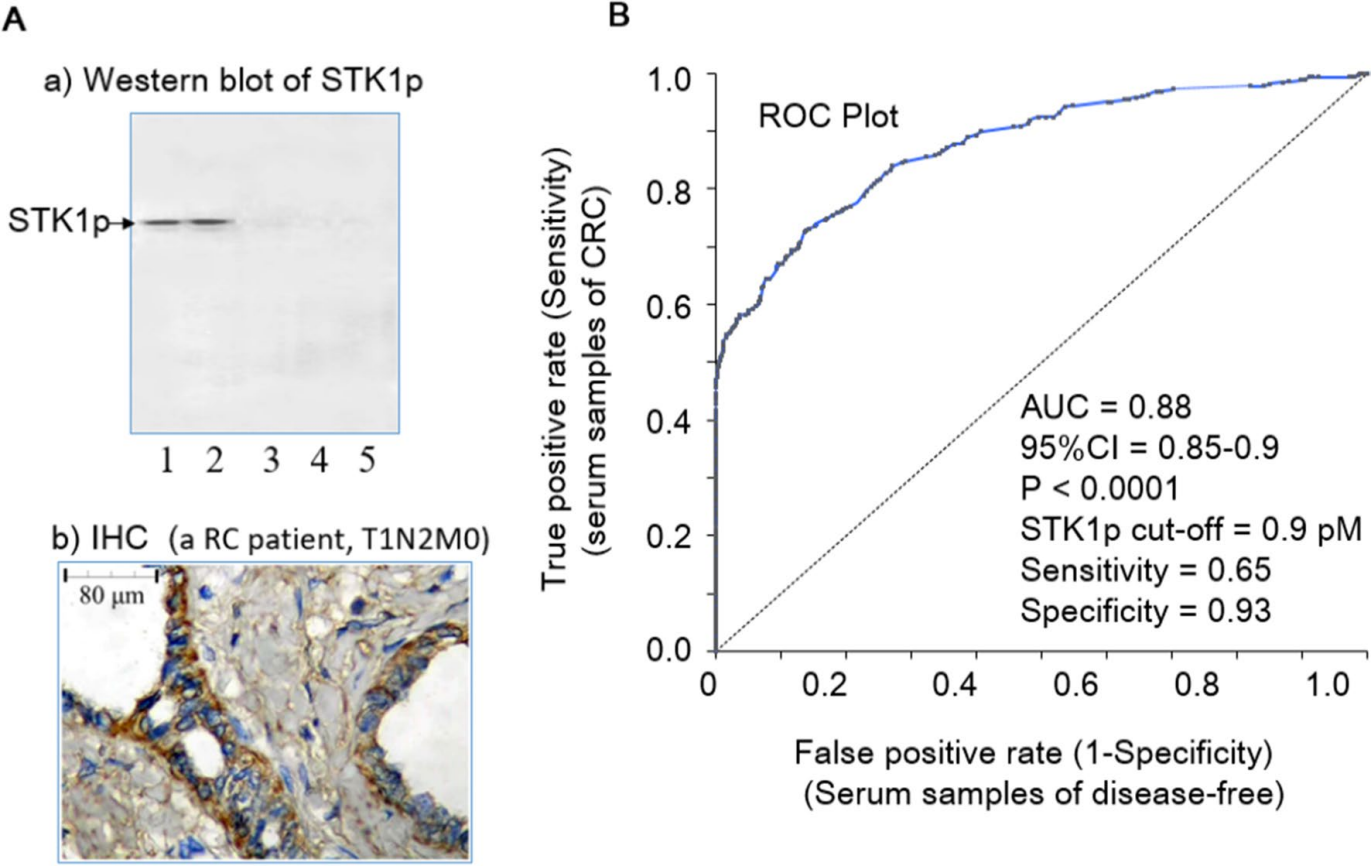
Characteristics of TK1-IgY-pAb. **A**. Example of a patient with RC (T1N2M0). **a** Western blot of STK1p. The serum samples, presurgery (Lines 1 and 2, duplicate), and 6 months after surgery (Line 3); Serum sample from a disease-free person (Lines 4 and 5, duplicate). b**)** TK1 immunohistochemistry (IHC) staining of RC tissue postsurgery (T1N2M0). Brownish-yellow TK1 was mainly in the cytoplasm. Blue staining was used to counterstain nuclei with hematoxylin. Magnification 200 × . **B**. Receiver operation characteristic (ROC) analysis. The analysis was based on STK1p values of 488 CRC patients and 488 tumor-free persons

The western blot of native–PAGE electrophoresis showed only one band corresponding to STK1p of RC patient presurgery (T1N2M0), and the level of STK1p at 6 months after surgery was reduced to an almost invisible band, similar to healthy controls (Fig. 1A-a). Strong staining of TK1 was found mainly in the cytoplasm of the tissue in RC (T1N2M0) patients (Fig. 1A, b), which is in agreement with previous studies of STK1p [17, 19]. The AUC value in the ROC statistical analysis was found to be 0.88. At the cutoff value of 0.88 (pmol/l, pM), the likelihood ( +) was 8.3, the sensitivity was 0.65, and the specificity was 0.93 (Fig. 1B). The high AUC and likelihood ( +) values showed that our STK1p assay is a reliable serum biomarker for CRC.

### STK1p values of R-CC, L-CC and RC patients

The STK1p values of R-CC, L-CC and RC patients were not significantly different (Table 1, P ≈ 0.39–0.64), except for a significant difference between the healthy people and the R-CC, L-CC and RC patients (Table 1, P < 0.001).

**Table 1:** STK1p values in PTL of CRC patients and disease-free persons. The PTL of CRC patients was divided into three different subgroups (1–1. R-CC; 1–2. L-CC;1–3. RC)

Group	Number	STK1p	p value
		mean ± SD	
1. CRC	488	1.84 ± 1.78	
Subgroups			
1–1. R-CC	90	1.91 ± 1.71	
1–2. L-CC	128	1.81 ± 1.80	
1–3. RC	270	1.92 ± 1.84	
2. Disease-free	488	0.42 ± 0.28	Subgroups vs. Disease-free:
			p < 0.001

### Kaplan‒Meier plots evaluated OS as a function of seven factors

OS was evaluated based on seven prognostic factors, including the serological biomarkers STK1p, CEA and CA19.9, as well as the clinical parameters TNM stage, pathological grade, age and sex.

Because there was no significant difference between TNM stages I and II or between pathological grades G1 and G2, we grouped them together.

#### OS of CRC

**Fig. 2:**
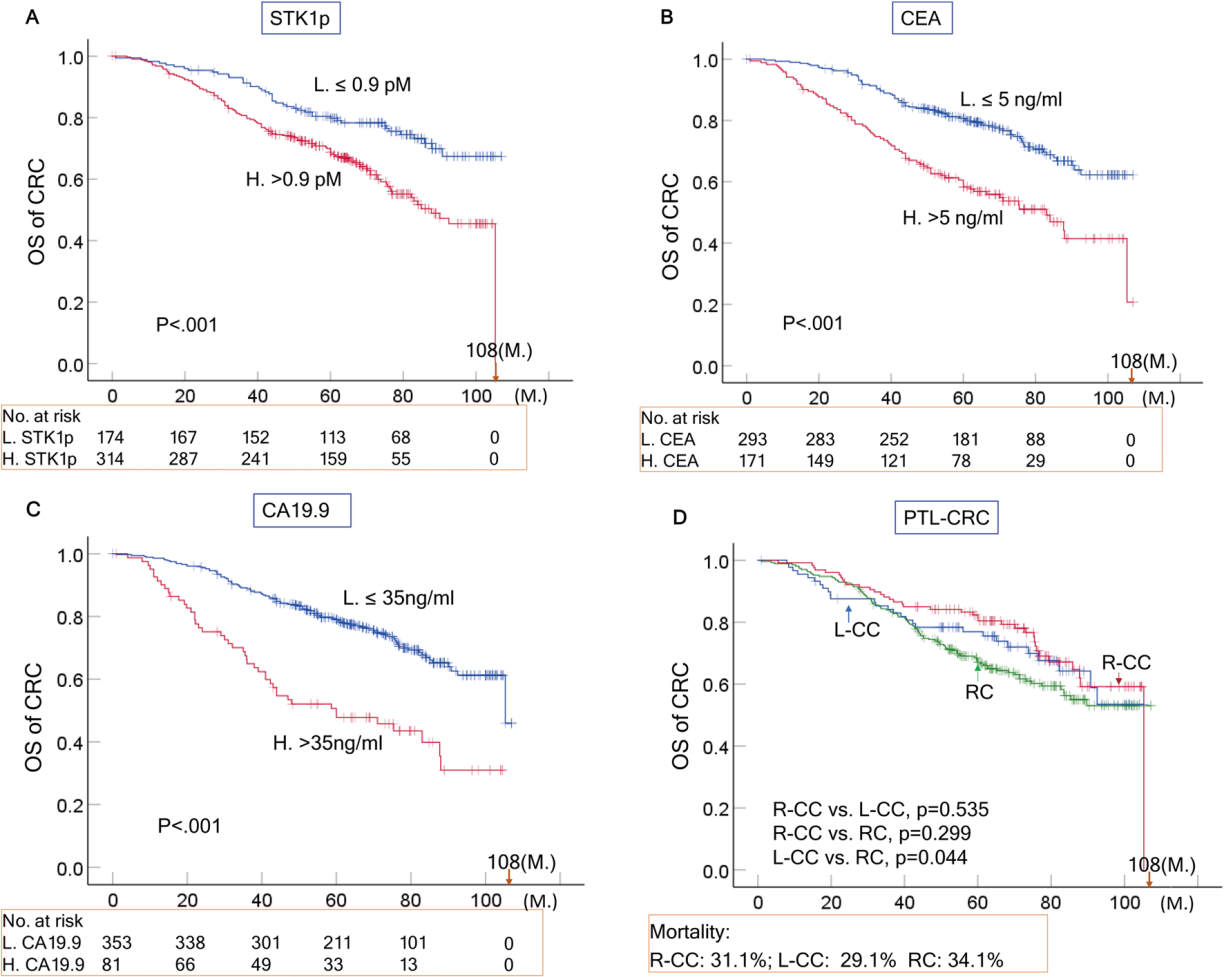
Overall survival (OS) rate of CRC patients related to STKIp, CEA and CA19.9 (**A**–**C**) and the OS rate related to PTL of CRC (**D**) based on the Kaplan–Meier plotter database. The solid dots in the survival curves show the times of censored observations. M: months

There was a statistically significant difference in OS of total CRC (n=488) based on the seven prognostic factors (Table 2A, P<0.05, Figs. 2A–C, P~0.000). However, when comparing the PTL of CRC groups with each other, no statistically significant difference in OS between R-CC (n=90) and L-CC (p=0.535, n=128) or between R-CC and RC (p=0.299, n=270) was found, while a statistically significant difference was found between L-CC and RC (p=0.044) (Fig. 2D). During the follow-up time of 108 months, the mortality values of the R-CC, L-CC and RC patients were 31.1%, 29.1% and 34.1%, respectively. No statistically significant difference was found (P>0.05).

**Table 2:** Kaplan‒Meier plots evaluating the OS of CRC and its PTL as a function of seven prognostic factors (STK1p, CEA, CA19.9, TNM stage, pathological grade, age and sex)

Item	Log-rank test (P-value)	X^2^
A. CRC		
1. STK1p, ≤ 0.9 vs. > 0.9 pM	**0.003**	**15.371**
2. CEA, ≤ 5 vs. > 5 ng/ml	**0.000**	**26.715**
3. CA19.9, ≤ 35 vs. > 35 ng/ml	**0.000**	**34.834**
4. TNM stage, I + II vs. III	**0.000**	**54.093**
5. Pathological grades, G1 + G2 vs. G3	**0.005**	**8.057**
6. Age, > 60 ≤ 60 ys.	**0.035**	**4.42**
7. Sex, F. vs. M	**0.033**	**4.566**
B. R-CC		
1. STK1p, ≤ 0.9 vs. > 0.9 pM	0.051	3.794
2. CEA, ≤ 5 vs. > 5 ng/ml	0.154	2.073
3. CA19.9, ≤ 35 vs. > 35 ng/ml	0.113	2.509
4. TNM stage, I + II vs. III	**0.006**	**7.476**
5. Pathological grades, G1 + G2 vs. G3	0.188	1.73
6. Age, > 60 ≤ 60 ys.	0.671	0.18
7. Sex, F. vs. M	0.667	0.185
C. L-CC		
1. STK1p, ≤ 0.9 vs. > 0.9 pM	**0.037**	**4.371**
2. CEA, ≤ 5 vs. > 5 ng/ml	0.054	3.726
3. CA19.9, ≤ 35 vs. > 35 ng/ml	0.067	3.352
4. TNM stage, I + II vs. III	**0.000**	**12.332**
5. Pathological grades, G1 + G2 vs. G3	0.256	1.241
6. Age, > 60 ≤ 60 ys.	**0.027**	**4.915**
7. Sex, F. vs. M	0.108	2.586
D. RC		
1. STK1p, ≤ 0.9 vs. > 0.9 pM	**0.002**	**9.401**
2. CEA, ≤ 5 vs. > 5 ng/ml	**0.000**	**25.189**
3. CA19.9, ≤ 35 vs. > 35 ng/ml	**0.000**	**38.996**
4. TNM stage, I + II vs. III	**0.000**	**34.554**
5. Pathological grades, G1 + G2 vs. G3	**0.03**	**8.708**
6. Age, > 60 ≤ 60 ys.	0.119	2.433
7. Sex, F. vs. M	0.156	2.01

Black bold: P-values of < 0.05 were considered statistically significant

#### OS of R-CC, L-CC and RC

**Fig. 3:**
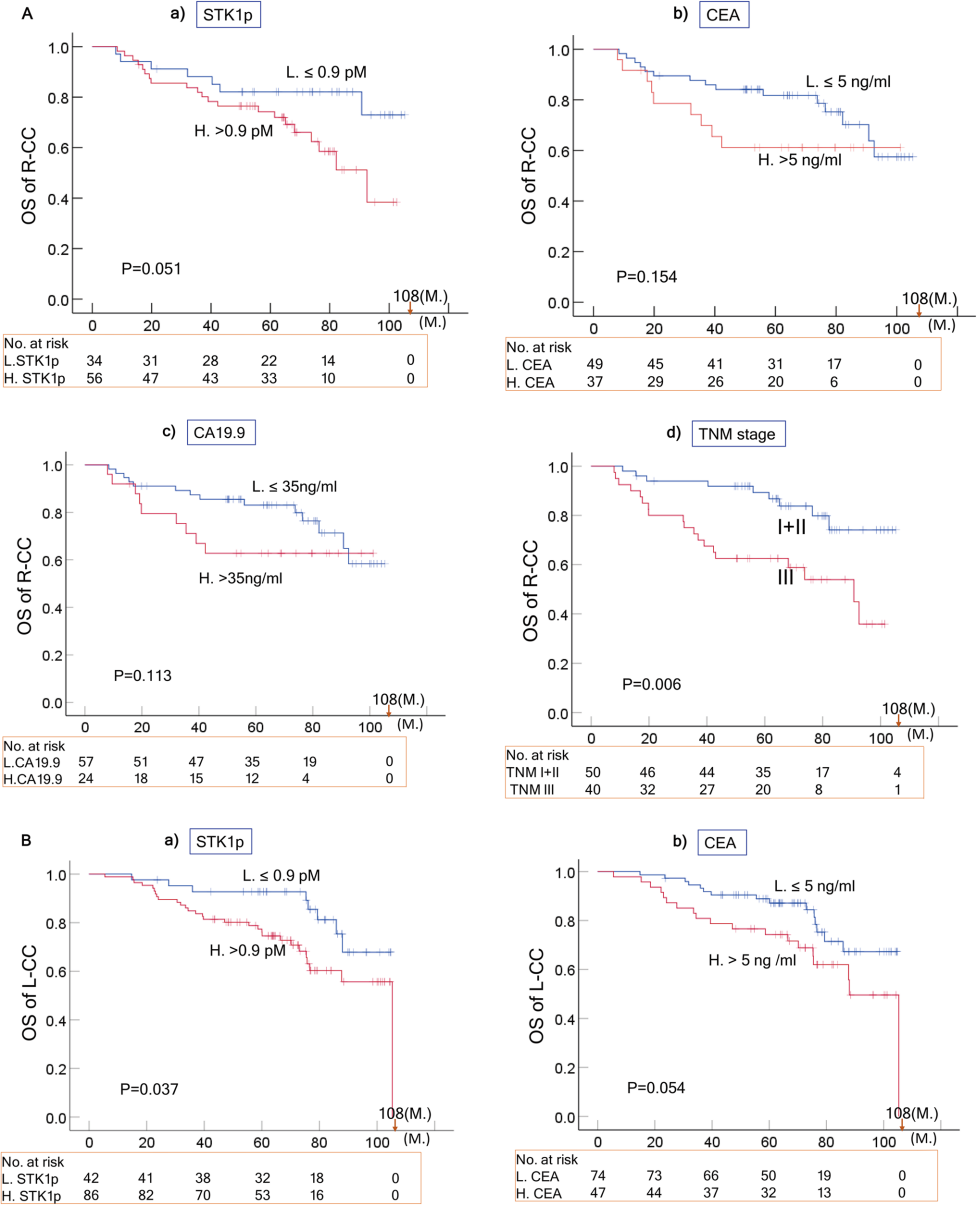

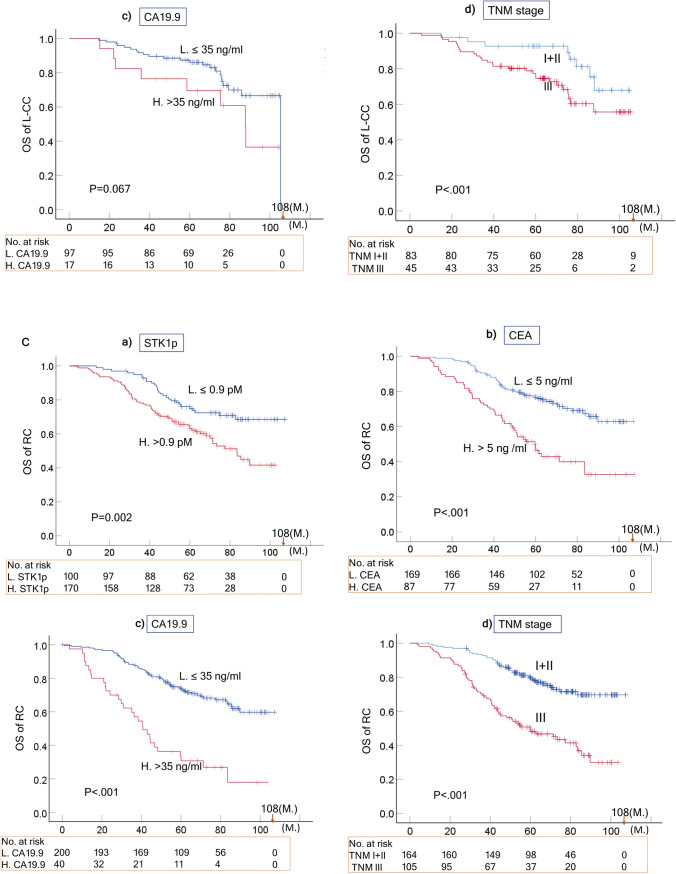
Overall survival (OS) curves of R-CC (**A**), L-CC (**B**) and RC (**C**) related to STK1p, CEA, CA19.9, and TNM stage based on the Kaplan–Meier plotter database. The solid dots in the survival curves show the times of censored observations. M: months

The Kaplan‒Meier plots evaluating the OS of the R-CC, L-CC and RC subgroups in relation to the seven prognostic factors are summarized in Tables 2B–D and shown in Fig. 3A–C. The TNM stage (I-II vs. III) significantly correlated with the OS of R-CC, L-CC and RC (p < 0.01). However, the other six prognostic factors (STK1p, CEA, CA19.9, pathological grade, age and sex) showed complex relationships.

##### R-CC

The STK1p was an almost significant prognostic biomarker (p = 0.051) in R-CC patients, based on the OS, while CEA and CA19.9 were not (Table 2B; Fig. 3A, P > 0.05).

##### L-CC

The STK1p (P = 0.037), and the ages (P = 0.027) were significant prognostic biomarkers in L-CC patients based on OS, but not CEA, CA19.9, pathological grades or sex (Table 2C; Fig. 3B, P > 0.05).

##### RC

The STK1p (P = 0.002), CEA (P ~ 0.000, CA19.9 (P ~ 0.000),and the pathological grade (P = 0.03) were significant prognostic biomarkers in RC patients based on OS, but not age or sex (Table 2D; Fig. 3B, P > 0.05).

### Sensitivity of elevated STK1p, CEA or CA19.9 for the risk of mortality

It is important for oncologists to monitor how effective their surgery/treatment is. One way to do that is to correlate elevated serum biomarkers to mortality, for example, number of deceased or increased mortality patients after treatment. In this study, elevated STK1p values at the end of the 108-month follow-up of the R-CC, L-CC and RC patients correlated with mortality rates of 75.0%, 75.7% and 71.6%, respectively (Table 3). The corresponding values for CEA and CA19.9 were 42.9%, 51.4%, 49.0% and 32.1%, 21.6%, 28.4%, respectively (Table 3). There was a statistically significant difference between STK1p and CA19.9 in all R-CC, L-CC and RC patients (P < 0.005).

**Table 3:** The elevated STK1p value at the end of the 108-month follow-up of the R-CC, L-CC and RC patients correlated with mortality. The p values of CEA and CA19.9 are in relation to STK1p, for percentage values or the number of the patients. Chi-sq.: chi-square test

Type	STK1p	CEA	CA19.9
	> 0.9 pM	> 5 ng/ml	> 35 ng/ml
R-CC	21/28 (75.0%)	12/28 (42.9%)	9/28 (32.1%)
Chi sq. p value		**0.018**	**0.0007**
L-CC	28/37 (75.7%)	19/37 (51.4%)	8/37 (21.6%)
Chi sq. p value		0.091	**0.000**
RC	73/102 (71.6%)	50/102 (49.0%)	29/102 (28.4%)
Chi sq. p value		0.103	**0.0003**

Black bold: P-values of < 0.05 were considered statistically significant

### Multivariate COX regression analysis

The prognostic factors STK1p, CEA, CA19.9, TNM stage, pathological grade, age and sex were used for the multivariate COX regression analysis.

#### CRC

The seven prognostic factors (STK1p, CEA, CA19.9, TNM stage, pathological grade, age and sex) were independent prognostic factors (Table 4A).

**Table 4:** COX multivariate analysis for OS of CRC (A), R-CC (B), L-CC (C) and RC (D)

COX multivariate analysis	Hazard risk	95% CI	p value
A. CRC			
STK1p, ≤ 0.9 vs. > 0.9 pM	1.828	1.242–2.691	**0.002**
CEA, ≤ 5 vs. > 5 ng/ml	1.539	1.539–1.102	**0.013**
CA19.9, ≤ 35 vs. > 35 ng/ml	2.474	1.664–3.677	**0.000**
TNM stage, I + II vs. III	2.839	2.893–2.024	**0.000**
Pathological grades, G1 + G2 vs. G3	1.68	1.128–2.503	**0.011**
Age, > 60 ≤ 60 ys.	1.666	1.171–2.370	**0.005**
Sex, F. vs. M.	0.675	0.463–0.982	**0.04**
B. R-CC			
TNM stage, I + II vs. III	3.139	1.30–7.582	**0.011**
C. L-CC			
TNM stage, I + II vs. III	4.168	1.980–8.852	**0.000**
D. RC			
STK1p, ≤ 0.9 vs. > 0.9 pM	1.777	1.094–2.753	**0.031**
CEA, ≤ 5 vs. > 5 ng/ml	2.716	1.796–4.382	**0.000**
CA19.9, ≤ 35 vs. > 35 ng/ml	2.119	1.293–3.472	**0.003**
TNM stage, I + II vs. III	2.716	1.796–4.382	**0.000**

Black bold: P-values of < 0.05 were considered statistically significant

#### R-CC

Only TNM stage was an independent prognostic marker (P = 0.011, Table 4B), and there was no statistical significance for the other factors (P > 0.05, data not shown).

#### L-CC

Only TNM stage was an independent prognostic marker (P < 0.001, Table 4C), and there was no statistical significance for the other factors (P > 0.05, data not shown).

#### RC

STK1p (P = 0.02), CEA (P = 0.001), CA19.9 (P.000) and TNM stage (P.000) were independent prognostic markers (Table 4D), and there was no statistical significance for the other factors (data not shown).

### Correlation between STK1p, CEA and CA19.9 values

**Fig. 4:**
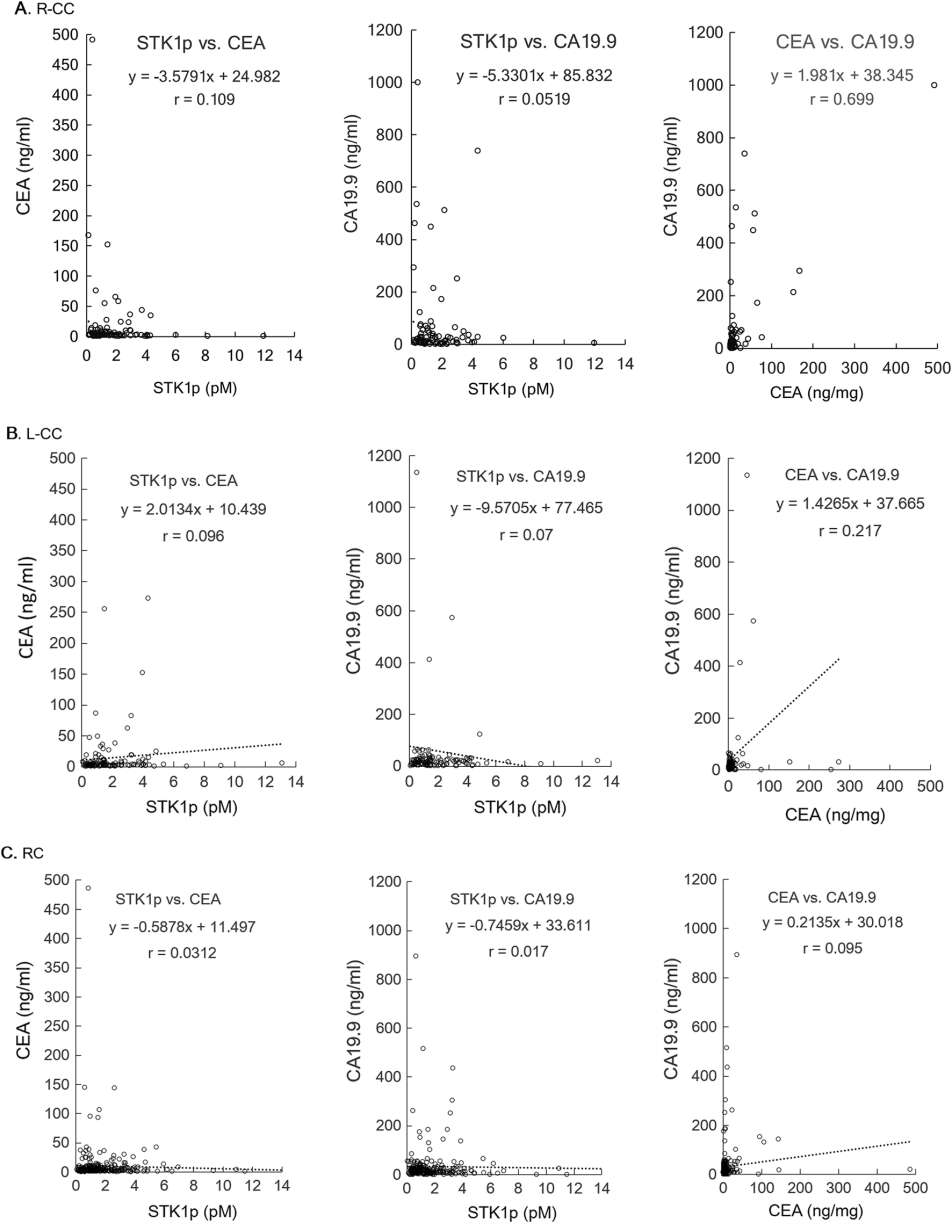
Correlation between serum values of STK1p, CEA and CA19.9 in R-CC (**A**), L-CC (**B**) and RC (**C**) patients. r = Pearson correlation coefficient

Serum biomarkers reflect different properties of a tumor, which may explain the discrepancies in the results between them [25]. There was no correlation between STK1p and CEA/CA19.9 or between CEA and CA19.9 in the CRC patients (r < 0.75, Figs. 4A–C).

## Discussion

In this clinical study, we investigated whether STK1p, CEA and CA19.9 can be used as prognostic factors in PTL (RLL, LCC and RC) of presurgery CRC patients. The usefulness as prognostic biomarkers was confirmed by multivariate COX regression analysis together with Kaplan‒Meier OS plots and determination of the sensitivity in relation to mortality%.

Of the seven prognostic factors investigated, three included serological biomarkers (STK1p, CEA, and CA19.9), and four clinical parameters (TNM stage, regional lymph node, pathological grade, age and sex) were found to have different statistical significance (Tables 2A-D, 3 and  4A-D; Figs. 2A–C, 3 A–C).

The obtained results may indicate the following:

1. A combination of STK1 with CA 19.9, CA 72–4 and CEA improves the evaluation of the treatment outcome of CRC patients [30]. In our study, the multivariate COX regression analysis showed that the seven prognostic factors (STK1p, CEA, CA19.9, TNM stage, pathological grade, age and sex) were independent prognostic factors (Table 4A). In addition, the seven prognostic factors also significantly correlated with survival (OS) (Table 2A, P < 0.05). However, the PTL of CRC shows differences in epidemiological, clinical and histological characteristics [6–10]. Combining all our results, COX analysis showed that TNM stage, STK1p, CEA and CA19.9 are independent prognostic factors in RC patients (Table 4). The OS rates of RC and L-CC were significantly associated with STK1p and TNM stage, except for R-CC (Table 2 and Fig. 3A–C). This finding supports the hypothesis that R-CC, L-CC and RC should be regarded as independent native entities (6). Searching for suitable serum biomarkers for the assessment of the outcome of R-CC should be necessary in further studies.
2. In our study, the standard antitumor therapy was oxaliplatin (see the treatment section). This treatment may be ineffective due to its lack of specificity for the PTL of CRC patients. The treatment is now improved by the development of new drugs such as platinum (Pt)- or other metal-based drugs such as gold (Au), silver (Ag), iridium (Ir), or ruthenium (Ru). However, the results of these new drugs are mostly based on animal studies [31]. Thus, curative surgical resection of primary CRC patients is still an important therapy. Our results also show that a low value of STK1p together with early TNM stage (I + II stage) is of benefit when assessing the prognosis of RC and L-CC patients, as described for breast [20, 21] and lung carcinoma [23].
3. There were no differences in the number of mortalities in R-CC, L-CC and RC patients (p > 0.05, Fig. 2D). However, elevated STK1p values were more sensitive to mortality% than CEA and CA19.9 (Table 3). High STK1p values were significantly associated with metastasis in lymph node/distant metastasis in patients with lung NSCLC of early/middle stages (IA-II) who had worse OS [23, 32]. This implies that elevated STK1p in primary presurgery patients serves as warning to doctors, indicating that the patient has a poor prognosis; thus, a reasonable treatment plan should be set up according to the individual medical situation of the patient to improve the survival efficiency of the patient.
4. The lack of a correlation between STK1p, CEA and CA19.9 (Fig. 4) can be explained by the fact that STK1p is specifically related to the proliferation rate of tumors, while CEA/CA19.9 is not, confirming previous results on STK1p, CEA and AFP [24]. The different tumor-related biomarkers have their own characteristics, which makes it difficult to evaluate the results when combining tumor-related markers. In addition, the Cigna Medical Coverage Policy mentioned in 2015 that tumor-associated antigens, such as CEA/CA19.9, can also be found in serum, plasma, urine, or other body fluids of normal healthy persons [33].

**Fig. 5:**
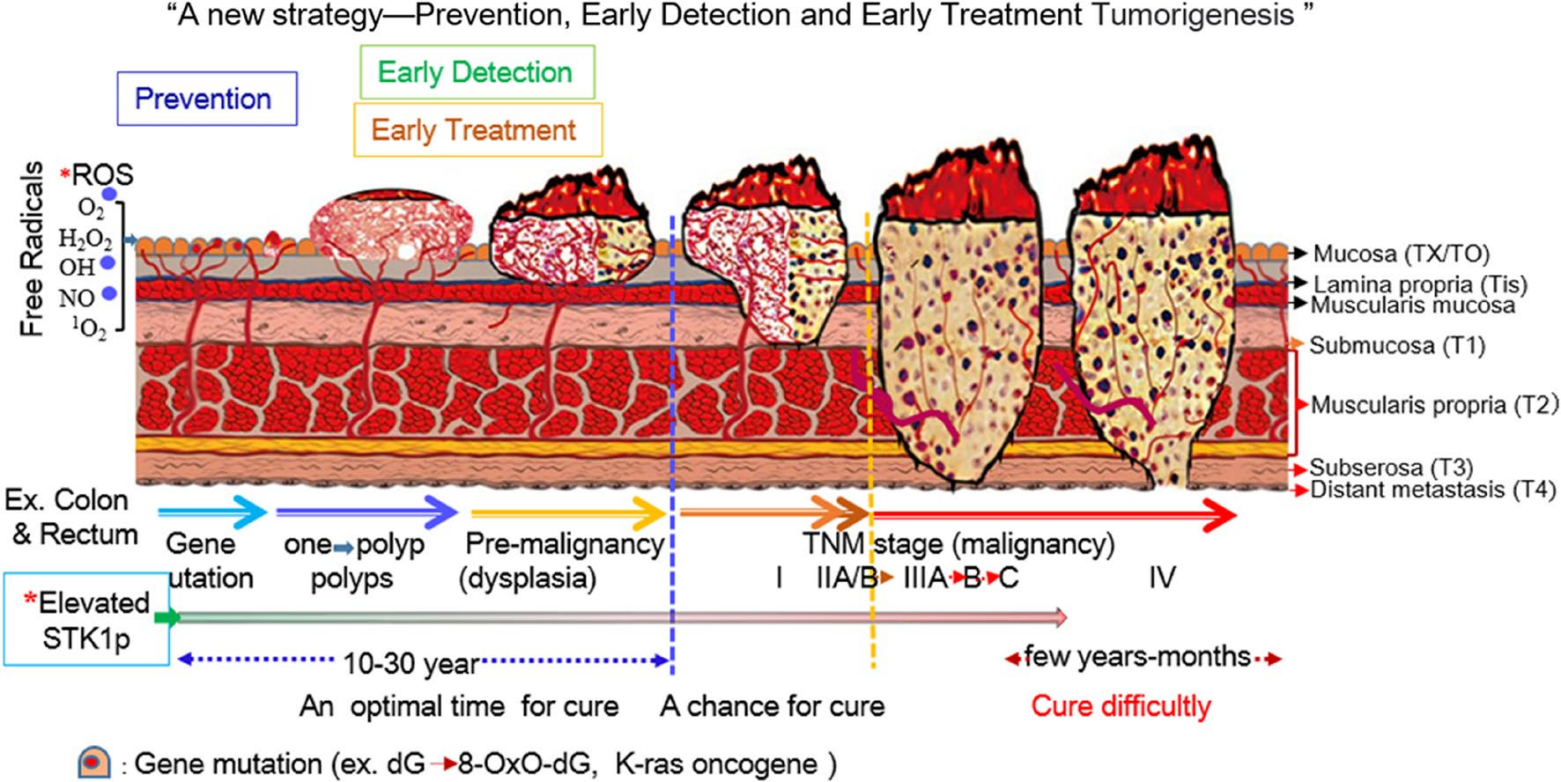
Schematic diagram of the detection of early colorectal tumorigenesis based on STK1p combined with appropriate imaging. ROS✸: Reactive oxygen species from metabolism, inflammation, radiation, pollution, etc.; *Elevated STK1p: the STK1p value significantly increased (P < 0.0001) in the following manner: healthy mucosa (tumor-free) < enlarged polyps < dysplasia < colorectal carcinoma (CRC, TNM stage I-III) [28]. The text is explained in reference no 30

Currently, clinical oncological studies are focused on the detection of precancerous lesions to enable early intervention in clinical oncology, preventing cancer progression and reducing mortality [34, 35]. This is important for a prerequisite successful treatment. In a recent report, it was shown that STK1p provided a reliable method that could discover invisible malignant tumors for early risk progression at the precancerous stage [36]. A meta-analysis of the STK1p value in colorectal adenoma polyp/dysplasia revealed that STK1p was significantly (p < 0.0001) distinguished among groups of tumor-free patients (n = 220) and patients with colorectal adenoma polyp (n = 271) and dysplasia (n = 198). CRC is a heterogeneous disease, ranging from healthy mucosa arising to benign polyp growth to dysplasia by the accumulation of genetic mutations and the progression of carcinogenesis [6]. The K-ras oncogene is a key factor and is the initial step in colorectal tumorigenesis. It can appear in benign polyps or in the normal accompanying mucosa polyps [37]. A strong recommendation from the European Society of Gastrointestinal Endoscopy (ESGE) Clinical Guideline is that all polyps should be resected except for diminutive (≤ 5 mm) rectal and rectosigmoid polyps that are predicted with high confidence to be hyperplastic [38]. We suggest detecting early colorectal tumorigenesis based on STK1p combined with K-ras mutations and appropriate imaging [28] and timely surgical treatment of patients, giving the best chance for a cure. A schematic diagram is presented in Fig. 5.

## Conclusion

Our study demonstrated for the first time that STK1p, a proliferation biomarker, is an independent prognostic factor of RC and is beneficial for the prediction of OS in patients with RC and L-CC. STK1p was a more reliable biomarker than CEA and CA19.9 concerning mortality in a routine clinical setting. We suggest that STK1p should be combined with TNM stage and specific serum biomarkers with high sensitivity for identifying early-stage precancerous lesions located in the L-CC, R-CC or RC, such as K–ras mutations and TK1 histochemical high staining of suspect polyps/precancerous lesions, to further improve the early prognosis of CRC-different entities patients.

## Research design

To assess whether STK1p, CEA and CA19.9 can be used as prognostic biomarkers in PTL of CRC. Four additional clinical factors, TNM stage, pathological grade, age and sex, were also included. **1.** The type of clinical study was a retrospective presurgery of R-CC (n = 90), L-CC (n = 128) and RC (n = 270) identified by histology. **2.** STK1p was measured by commercial enhanced chemiluminescence (ECL) dot blot enzymatic immunoassay kits. CEA and CA19.9 were determined using a commercial automatic electrochemiluminescence analyzer. **3.** The prognostic factors were evaluated by Kaplan‒Meier overall survival (OS) plots, multivariate COX regression analysis and determination of the sensitivity of these elevated biomarkers in relation to mortality. **4**. Establish a suitable risk threshold values of STK1p, CEA and CA-19.9 for the evaluation of prognostic factors.

## Participants

### Patients

A retrospective study of 488 primary CRC patients, including R-CC (n = 90), L-CC (n = 128) and RC (n = 270) patients, was performed between May 2004 and February 2012, and their data and samples were stored until analysis at the Sun Yat-sen University Cancer Centre, Guangdong, P.R. China. All serum samples were collected the day before surgery and stored at − 20 °C until analysis in July 2013. All samples were selected from a cohort of 8314 patients and histologically confirmed. The TNM staging criteria followed the AJCC cancer staging manual [39]. Cases with known hereditary nonpolyposis CRC, familial adenomatous polyposis, and a previous history of malignancy were excluded.

The relevant information, including age, sex, clinical stage, number of lymph nodes, histological type, chemotherapy, values of serum biomarkers (such as STK1p, CA19.9 and CEA), and survival time (death or alive) of each patient were extracted from the computerized hospital information system. The characteristics of the patients’ information are summarized in Table 5.

**Table 5:** PTL characteristics of CRC patients

Type	R-CC (n = 90)	L-CC (n = 128)	RC (n = 270)	Total (n = 488)
Age (ys.)				
≤ 60	49	58	139	246
> 60	41	70	130	241
No info			1	1
**≦60/ > 60**	**49/41**	**58/70**	**139/130**	**264/241**
Gender				
M	55	78	162	295
F	35	50	108	193
No info				
** Gender, M/F**	**55/35**	**78/50**	**162/108**	**295/193**
TNM stage				
I	14	21	78	113
IIA	18	33	44	95
IIB	18	29	42	89
IIIA	3	3	8	14
IIIB	24	32	55	111
IIIC	13	10	42	65
No info			1	1
**I + II/III**	**50/40**	**83/45**	**164/105**	**297/190**
Histological grade				
G1	3	9	17	29
G2	62	96	206	364
G3	23	22	45	90
No info	2	1	2	5
G1 + G2/G3	65/23	105/22	223/45	393/89
STK1p, cutoff				
≤ 0.9 pM	34	42	100	176
> 0.9 pM	56	86	170	312
** ≤ 0.9/ > 0.9 pM**	**34/56**	**42/86**	**100/170**	**176/312**
CEA, cutoff				
≤ 5 ng/ml	49	74	169	292
> 5 ng/ml	37	47	87	171
No info	4	7	14	25
** ≤ 5/ > 5 ng/ml**	**49/37**	**74/47**	**168/88**	**292/171**
CA19.9, cutoff				
≤ 35 ng/ml	57	97	220	352
> 35 ng/ml	24	17	40	82
No inf	9	14	10	54
** ≤ 35/ > 35 ng/ml**	**57/24**	**97/17**	**199/40**	**352/82**

### Treatment

All patients had undergone curative surgical resection followed by standard routine adjuvant chemotherapy, according to the recommendations for CRC patients [40, 41]. Briefly, the patients were treated with one of three different regimens: 1. The FOLFOX_6_ program (oxaliplatin 100 mg/m^2^, CF 400 mg/m^2^ and 5-Fu 2.4–3.0 g/m^2^) was administered by intravenous injection for 2 weeks/2 cycles; 2. The XELOX program (intravenous injection with oxaliplatin 135 mg/m^2^) and 3. The XELODA program (2,500 mg/m^2^) were orally administered (3 weeks/3 cycles).

### Follow-up

The time of follow-up was 108 months. The median was 63.78 months. The survival status of the 488 cases was obtained from the medical records of the clinical department by telephone or by written contact with the patients.

## Methods

### Characteristics of TK1-IgY-pAb

Serum samples and tissue from an RC patient (T1N2M0) were used as examples to determine whether the TK1-IgY-pAbs were specific and sensitive as tumor proliferating biomarkers using native–PAGE electrophoresis, western blot and TK1 immunohistochemistry (IHC) staining [21, 22]. The serum samples were stored in our laboratory at − 80 °C for 2 years. The immunohistochemistry was conducted as described by Skog et al., 2017 [17] with slight modifications. Briefly, Sections. (4-μm-thick) were prepared from formalin-fixed paraffin-embedded surgical specimens from the RC patient by deparaffinization and rehydration and then incubated with TK1-IgY-pAb (1.0 µg/ml, in PBS) overnight at 4 °C followed by incubation with biotinylated donkey anti-chicken IgY antibody at room temperature for 60 min. SA-HRP (streptavidin-HRP conjugate, Invitrogen, SA10001) was added and incubated at room temperature for 90 min. Fresh diaminobenzidine (DAB) solution was used for color rendering, and the slides were lightly counterstained with hematoxylin.

### Assay of STK1p

The serum samples were stored at − 20 °C until analysis in July 2013. The mean storage time for the 488 serum samples was 8.87–107.23 months. The stability of TK1 in the serum was investigated by measuring TK1 activity after incubation at 56 °C for 30 min, and the TK1 activity was maintained at 70% [42]. The reason for the extensive stability is that TK1 in serum is in a large complex with proteins consisting of a ≈730 kD complex linked by S‒S bridges to other proteins [43]. The stability of TK1 in serum during storage for 10 years at − 20 °C was also investigated. The value of the TK1 concentration was more than 85% after 10 years of storage at − 20 °C [28]. Thus, TK1 is stable for at least 10 years at − 20 °C.

### Serum thymidine kinase 1 protein concentration (STK1p) assay

The STK1p was measured by a commercial enhanced chemiluminescence (ECL) dot blot kit based on TK1-IgY-pAb (Sino-Swed Tong Kang Bio-Tech, Ltd., Shenzhen, China, http://www.sstkbiotech.com). The controls were disease-free subjects (n = 488, between 19 and 89 years old; median age 53 years old, men 54 years old, women 52 years old) who did not show any symptoms of malignancies or cancer-related diseases, infection or inflammatory diseases. The controls were collected during 2005–2011 at the Healthy Centre of the Third XiangYa Hospital, University, ChangSha, China.

### Measurement of serum CEA and CA19.9 levels

The serum levels of CEA and CA19.9 were determined using enzymatic immunoassay kits (Cobas^®^602, Roche, Diagnostics, Mannheim, Germany) by an automatic electrochemiluminescence analyzer (E170, Roche, Germany). According to previous studies of preoperative serum levels of CEA and CA 19.9 for prognostic significance in CRC, a threshold value of 5 ng/ml of CEA [44] and 35 ng/ml of CA 19.9 [45] were recommended. We performed an investigation using our own serum samples and confirmed that a suitable threshold value of 5 ng/ml was for CEA and 35 ng/ml for CA 19.9 for our study.

## Variables and definitions

The outcome of interest was overall survival (OS). The survival interval was defined as the time from histological diagnosis to death from any cause or the end of follow-up.

## STK1p cutoff

There is no prior report of the preoperative serum levels of STK1p and its prognostic significance in CRC. The receiver operating characteristic (ROC) analysis in this study was important, providing information on how reliable a test is, defined by its area under the curve (AUC) and its likelihood ( +) value. STK1p values below 0.9 pM were denoted as “low STK1p”, and STK1p values above 0.9 pM were denoted as “elevated STK1p” in the analysis of the overall survival (OS) rate and the COX analysis.

## Statistical analysis

Statistical significance was determined using the statistical program SPSS 25.0 (V25.0, IBM, USA), including evaluation of prognostic factors by Kaplan‑Meier plot and multivariate COX regression models. Between the parameters, significance was analyzed by correlation-Pearson test, chi-square test, T-test, the mean ± standard deviation and analysis of variance (ANOVA). Analysis-it (UK) was used for the ROC plot of STK1p. An r-value > 0.75 was considered to be a significant correlation. P-values of < 0.05 were considered statistically significant.

## Data Availability

All data generated or analyzed during this study are included in this article.

## Abbreviations

AUC: Area under the curve
CA19.9: Carbohydrate antigen
CEA: Carcinoembryonic antigen
COX: Proportional hazard model
OS: Overall survival
DFS: Disease-free survival
dTTP: Deoxy-thymidine-tri-phosphate
ECL dot blot: Enhanced chemiluminescence dot bot
GLOBOCAN: Global cancer observatory
HTK1: Human thymidine kinase 1
IUAC: International Union Against Cancer
L-CC: Left colon carcinoma
NACB: National Academy of Clinical Biochemistry
PAGE: Polyacrylamide gel
PTL: Primary tumor location
RC: Rectal carcinoma
R-CC: Right colon carcinoma
ROC: Receiver operating characteristic analysis
STK1p: Concentration of serum thymidine kinase 1
TK1 IgY-pAb: Chicken anti-human TK1 IgY poly-antibody
TNM: Tumor size, lymphatic node and metastasis
UICC: Union for International Cancer Control
